# Podoplanin is Responsible for the Distinct Blood and Lymphatic Capillaries

**DOI:** 10.1007/s12195-022-00730-2

**Published:** 2022-08-06

**Authors:** Donghyun Paul Jeong, Eva Hall, Erin Neu, Donny Hanjaya-Putra

**Affiliations:** 1 Department of Aerospace and Mechanical Engineering, Bioengineering Graduate Program, University of Notre Dame, 141 Multidisciplinary Research Building, Notre Dame, IN 46556 USA; 2 Department of Chemical and Biomolecular Engineering, University of Notre Dame, Notre Dame, IN 46556 USA; 3 Harper Cancer Research Institute, University of Notre Dame, Notre Dame, IN 46556 USA

**Keywords:** Blood endothelial cells, Lymphatic endothelial cells, Fibrin hydrogels, Podoplanin, Blood-lymphatic separation

## Abstract

**Introduction:**

Controlling the formation of blood and lymphatic vasculatures is crucial for engineered tissues. Although the lymphatic vessels originate from embryonic blood vessels, the two retain functional and physiological differences even as they develop in the vicinity of each other. This suggests that there is a previously unknown molecular mechanism by which blood (BECs) and lymphatic endothelial cells (LECs) recognize each other and coordinate to generate distinct capillary networks.

**Methods:**

We utilized Matrigel and fibrin assays to determine how cord-like structures (CLS) can be controlled by altering LEC and BEC identity through podoplanin (*PDPN*) and folliculin (*FLCN*) expressions. We generated BEC^*ΔFLCN*^ and LEC^*ΔPDPN*^, and observed cell migration to characterize loss lymphatic and blood characteristics due to respective knockouts.

**Results:**

We observed that LECs and BECs form distinct CLS in Matrigel and fibrin gels despite being cultured in close proximity with each other. We confirmed that the LECs and BECs do not recognize each other through paracrine signaling, as proliferation and migration of both cells were unaffected by paracrine signals. On the other hand, we found *PDPN* to be the key surface protein that is responsible for LEC-BEC recognition, and LECs lacking *PDPN* became pseudo-BECs and vice versa. We also found that *FLCN* maintains BEC identity through downregulation of *PDPN*.

**Conclusions:**

Overall, these observations reveal a new molecular pathway through which LECs and BECs form distinct CLS through physical contact by *PDPN* which in turn is regulated by *FLCN*, which has important implications toward designing functional engineered tissues.

**Supplementary Information:**

The online version contains supplementary material available at 10.1007/s12195-022-00730-2.

## Introduction

The lymphatic system is an essential secondary vascular system that is responsible for key functions such as interstitial pressure regulation, immune cell trafficking, and dietary fat absorption.^15,43^ Damages to lymphatic vessels are associated with lymphedema, cancer metastasis, and inflammation, showing the importance of lymphatic system to proper tissue function.^4,5^ Despite its significance, the lymphatic system has only been a subject of investigation with the discovery of markers in the last 20 years, including podoplanin (PDPN), lymphatic vessel endothelial hyaluronan receptor-1 (LYVE-1), prospero-homeobox-1 (Prox1) that distinguish lymphatic endothelial cells (LECs) from that of blood endothelial cells (BECs).^7,27,55,58^

Discovering the molecular mechanism that controls angiogenesis and lymphangiogenesis is crucial for the future of tissue engineering.^3,21,28,36^ Due to limitations of nutrient diffusion, engineered tissues with thickness in any dimension exceeding 400 *μ*m require a vascular system for growth and survival after *in vivo* implantation.^6^ While most of the research into vascularized tissue engineering has been focused on blood vessels,^19,22,33^ the addition of lymphatic vessels to engineered tissues has been shown to impart immunological functions to organs and improve their functions.^2,36,37,51^ It has been demonstrated that in vascular organoids and tissue engineered skin grafts, blood and lymphatic vessels do not form joined microvasculature.^1,36^ Physiologically, venous and lymphatic vessels use different valve systems, where the venous valve contracts but lymphatic valves contract rhythmically to pump the lymph.^11,26,39^ These incompatibilities indicate that the two cell lines maintain separation and undergo distinct capillary tube formation,^25,49,54^ but the exact molecular mechanism behind BECs and LECs recognition is yet unclear.

According to the widely accepted venous origin theory, LECs originate from the cardinal vein during embryonic development when venous endothelial cells express adult lymphatic marker LYVE-1 and PDPN.^8,42^ The committed LECs express lymphatic markers and master regulator gene *Prox1*, which lead to subsequent divergence of cell lines, but mutations in LEC-determinant genes can lead to mispatterning and lymphatic and blood vessels mixing.^27,44,46^ Following lymphatic commitment, the two vessels do not normally form conjoined vessels, but undergo separate capillary tube formation despite developing in the vicinity of each other.^30,48^ When BECs and LECs are cultured in a fibrin scaffold, they form separate, distinguishable networks.^23,31^ Recently, a tumor suppressor gene called folliculin (*FLCN*) has been identified as a key regulator in maintaining LEC-BEC separation by inhibiting *Prox1* expression in BECs, which may suppress expression of other lymphatic markers such as *PDPN*.^54^ Inhibiting *FLCN* in BECs causes them to express some LEC-like features and lead to the formation of blood-filled lymphatic vessels. Similarly, inhibition of a transmembrane protein called PDPN, one of lymphatic markers responsible for early separation process of LECs from BECs, also results in blood-filled lymphatic vessels in mice.^9,10,18,55^

Here, we identify a novel pathway that regulates distinct cord-like networks formation through cell–cell recognition between LEC and BEC. This finding, along with a better understanding of how LECs and BECs interact with different biomaterials, will allow us to exert greater control over development of tissue engineered tissues with functional blood and lymphatic vessels.

## Materials and Methods

### Human BEC and LEC Culture

Human BECs and LECs derived from the dermis of two adult donors (PromoCell, Heidelberg, Germany) were expanded and used for experiments between passages 5 and 9. Human LECs were grown in endothelial cell growth medium MV 2 (EGM MV2; PromoCell) incubated at 37 °C with 5% CO_2_. Human BECs were characterized for the positive expression of CD31 and for the negative expression of Prox-1 and PDPN. Human LECs were characterized for the positive expression of CD31, Prox-1, and PDPN throughout the experiments. All cell lines were routinely tested for mycoplasma contamination and were negative throughout this study.

### Migration Assay

Cell migration was examined using the transwell assay. Transwell inserts (Falcon™ Cell Culture Inserts 08-771-21, pore size 8 *µ*m) were placed into a 24-well plate and pre-coated with collagen I. Collagen-coated transwells were washed with PBS and allowed to air-dry in the biosafety hood. 30,000 cells per well were seeded in the top portion of the transwell insert. Migration was stimulated by a 10% FBS gradient added to the bottom of the wells. The transwell plate was incubated at 37 °C, 5% CO_2_. After a 4 h incubation period, the top part of each transwell was wiped with a cotton swab to remove non-migrated cells. Migrated cells at the bottom of the transwells were fixed with 4% paraformaldehyde for 15 min at 37 °C, then washed with PBS. Fixed cells were stained with 1% Crystal Violet in 10% Acetic Acid at room temperature for 10–15 min, then washed with PBS. Seven to eight random areas were imaged per condition and quantified to determine the number of migrated cells in the assay.

### Wound Healing Assay

The 2-well culture inserts (ibidi) were placed in each well of a 24-well plate precoated with rat-tail collagen Type 1 solution (50 *µ*g/mL, Corning). Cells were seeded inside each chamber and incubated for 24 h to reach confluence. At this point, the culture inserts were removed to create scratch areas and imaging was initiated (Lionheart FX Automated Microscope, BioTek) to visualize the wound closure process. Wound confluency was measured in 30 min increments for 14 h. Data was obtained from Gen5 software (BioTek) and was analyzed using GraphPad Prism.

### 2D Matrigel Assay

To visualize network formation *in vitro*, a Matrigel angiogenesis assay was performed using a 15-well angiogenesis plate (*µ*-Slide Angiogenesis, ibidi).^57^ Each well was coated with Matrigel and incubated at 37 °C for at least 2 h. Cells were then seeded onto each Matrigel-containing well at a density of 4,000 cells per well. Network formation was visualized and imaged every 30 min for 10 h (Lionheart FX Automated Microscope, BioTek). Analysis was performed using AutoTube, an open source MATLAB software.^50,56^ Data was analyzed using GraphPad Prism.

### 3D Vasculogenesis Assay

The first layer of fibrin gel was prepared by mixing 30 *μ*L of 7 wt% fibrinogen solution with 20 *μ*L of thrombin solution provided by Fibrin *In Vitro* Angiogenesis Assay kit (Sigma Aldrich) into each of 96-well plate wells and incubating at 37 °C for 20 min. Relevant cells were passaged using trypsin/EDTA solution and resuspended into fresh MV2 media containing 100 ng/mL of VEGF-C at concentration of 50,000 cells/mL. 100 *μ*L of the cell suspension was added on top of the first layer of the gel and allowed to settle for 24 h at 37 °C. Then the media was aspirated and another layer of 30 *μ*L of fibrinogen and 20 *μ*L of thrombin solution was added on top of the seeded cells and incubated at 37 °C for 5 min. Then 100 *μ*L of MV2 media containing 100 ng/mL of VEGF-C was added to top of the gel. Cells were allowed to form networks for 48 h, then imaged. Fibrin gels were imaged on the confocal microscope (A1R Nikon) using Texas Red and FITC channels. We captured 31 z-stack images across 200 *μ*m along the *z*-axis centered around the stack with the brightest fluorescence signals as determined visually.

### Angiogenesis Assay in Microfluidic Device

To mimic blood and lymphatic sprouting into 3D matrices, we performed angiogenesis assay using IdenTX chip (AIM Biotech, Singapore) following manufacture protocol and previous studies.^24,29^ Briefly, the middle channel was filled with fibrin gel by mixing 6 *μ*L of 7 wt% fibrinogen solution with 4 *μ*L of thrombin solution provided by Fibrin *In vitro* Angiogenesis Assay kit (Sigma Aldrich). To induce angiogenesis, 2 *μ*M of S1P (Sphingosine-1 phosphate) and 100 ng/mL of VEGF-C were encapsulated into the fibrin gel.^22^ After gelation, an equal density of BEC and/or LEC (5 × 10^6^ cells/mL) were seeded on each chamber of the IdenTx Chips separated by the fibrin gel in the middle. After 24 h, BEC and/or LEC were found invading the fibrin gel. The two channels system enabled easy access to the fibrin gel region for angiogenesis study with BEC and/or LEC.

### Microvasculature Network Quantification Method

We quantified the vascular network using AutoTube, an open source MATLAB software that can process a fluorescent image and generate skeletonized outline of the network.^38^ For each image, we analyzed each fluorescent channel separately and performed a max-projection across all z steps and adjusted the contrast such that the cells were clearly visible with low background signal. Ten parameters per image were quantified, and the tubes/node ratio and network area were selected to compare the degree of CLS formation on each substrate. For each hydrogel condition, at least three independent experiments were performed with two technical replicates.

### RNAi Transfection

Human BECs or LECs were transfected with siGENOME SMARTpool human *FLCN* or human *PDPN* (Dharmacon, Lafayette, CO) using the manufacturer’s protocol. Human BECs or LECs were cultured to 90% confluency in 6-well plates with EGM MV2 media (PromoCell) and no additional VEGF-C supplementation. The RNAi transfection solution was prepared by mixing DharmaFECT2 RNAi transfection reagent (Dharmacon) with serum-free and antibiotic-free EGM MV2 media. To transfect the cells, EGM MV2 media was removed and replaced with 1.6 mL of antibiotic-free EGM MV2 and 400 *µ*L transfection solution in each well to achieve a final RNAi concentration of 50 nM. Transfected cells were incubated at 37 °C and 5% CO_2_. After 72 h, total RNA was isolated and real-time qRT-PCR was performed, as described in the previous sub-section, to confirm the knock-down of *FLCN* or *PDPN* expression.

### Gene Expression

To analyze the gene expressions, BECs or LECs were cultured on hydrogels or tissue culture plastic for 48 h in their culture media. The 48 h timepoint was selected to ensure that the signaling cascade in response to VEGF-C and mechanical stimulation was captured. Each biological replicate was created by pooling RNA from three individual wells to collect enough RNA. At least three biological replicates (*n* = 3) were collected per condition and analyzed with real-time qRT-PCR with triplicate readings as previously described.^25,49^ RNA was reverse transcribed using a High-Capacity cDNA Reverse Transcription Kit (Thermo Fisher) according to the manufacturer’s protocol. cDNA was then used with the TaqMan Universal PCR Master Mix and Gene Expression Assays for *PDPN, FLCN,* and *GAPDH*. Each sample was prepared in triplicate and the relative expression was normalized to *GAPDH* and analyzed using the ^ΔΔ^*Ct* method.

### Statistical Analysis

Data are presented as mean ± standard deviation, unless otherwise were specified in the figure legends. All statistical analysis was conducted in GraphPad Prism. Statistical comparisons were made using Student’s *t* test for paired data, analysis of variance (ANOVA) for multiple comparisons, and with Tukey *post hoc* analysis for parametric data. Significance levels were *set* at the following: *p < 0.05, **p < 0.01, ***p < 0.001, ****p < 0.0001.

## Results

### Podoplanin is Uniquely Expressed by LECs, But Not BECs

**Figure 1:**
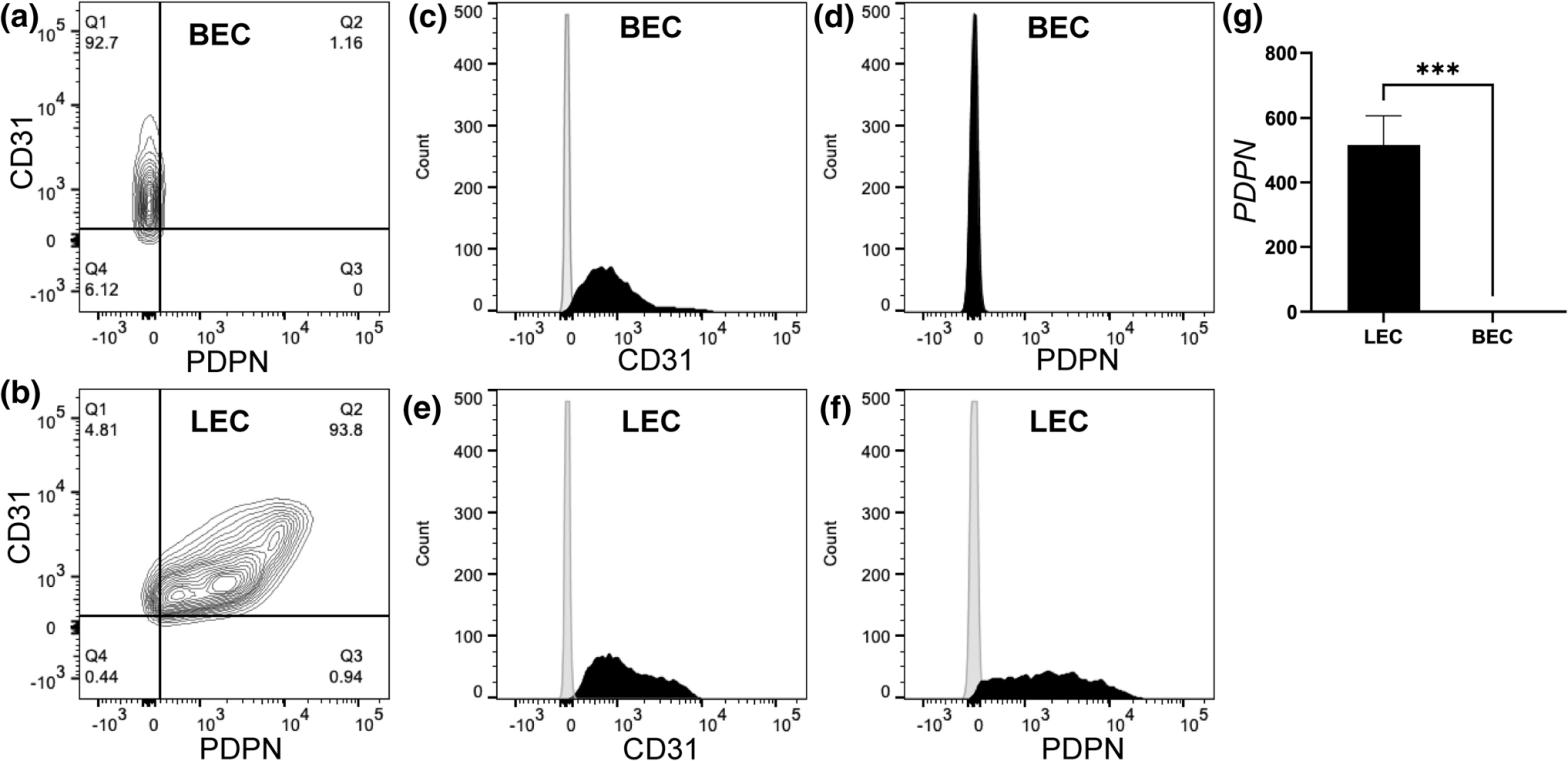
BECs and LECs express unique endothelial surface markers. Representative flow cytometry diagrams demonstrating unique endothelial surface markers for PDPN and CD31. (a) BEC (92.7%) showing CD31^+^ and PDPN^−^, while (b) LEC showing CD31^+^ and PDPN^+^. Representative flow cytometry histograms indicating (c and d) BEC and (e and f) LEC stained with CD31-PE (in *black*), PDPN-FITC (in *black*), and isotype controls (in *grey*). (g) Real-time quantitative qRT-PCR was used to analyze *PDPN* expression for LEC and BEC relative to *GAPDH*. Data represents mean $\pm$ SD, *n* = 4 per group, ***p < 0.001. All *p* values were determined by unpaired *t* tests.

Since both BECs and LECs were isolated from dermal skin vasculatures,^3,21^ the initial study was done to first characterize the unique surface markers specific to blood and lymphatic vasculatures. Flow cytometry analysis confirmed that BECs express CD31, but not Podoplanin (Fig. 1a and Supplementary Fig. 1A). On the other hand, LECs expressed both CD31 and Podoplanin (Fig. 1b and Supplementary Fig. 1B). Further quantification of flow cytometry histograms indicated that 92.7% of BECs were CD31^+^ and PDPN^−^ (Figs. 1c and 1d), while 93.8% of LECs were CD31^+^ and PDPN^−^ (Figs. 1e and 1f). These data suggests that the BECs and LECs were pure population of blood and lymphatic endothelial cells, respectively.

### BECs and LECs Form Distinct Cord-Like Structures

**Figure 2:**
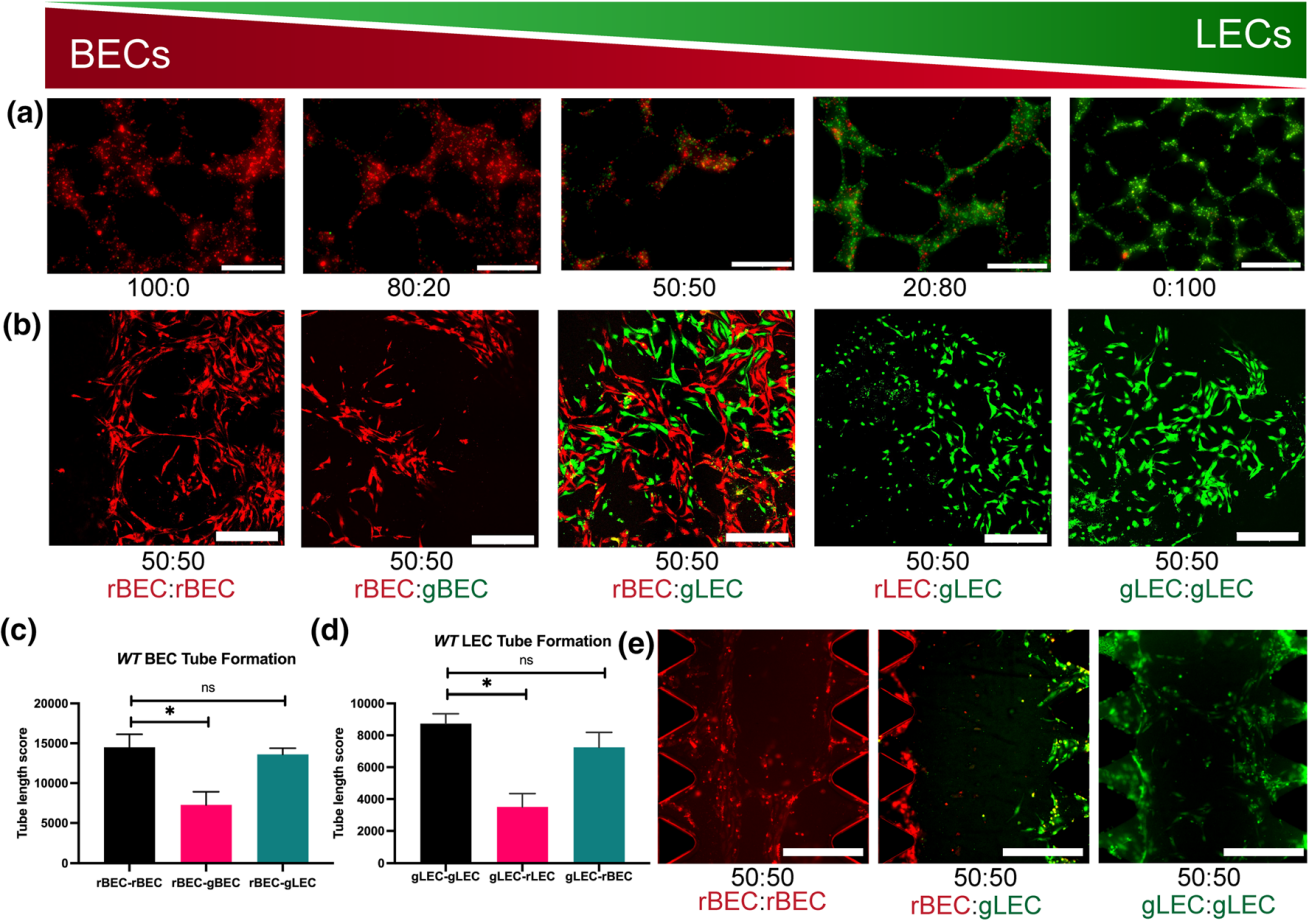
BEC and LEC form distinct cord-like structures on 2D Matrigel and 3D fibrin gel assays. (a) BEC (pre-labeled in pre-labeled in CellTracker™ Red CMTPX) and LEC (pre-labeled in CellTracker™ Green CMFDA) were seeded on 2D Matrigel at ratios of 100:0, 80:20, 50:50, 20:80, and 0:100 (BECs:LECs). Representative images of cord-like structures (CLS) formation were imaged at 12 h. Scale bars are 500 *μ*m. (b) BEC and LEC were encapsulated in 3D fibrin gel assay. From left to right, red-BEC only, red-BEC and green-BEC (50:50), red-BEC and green-LEC (50:50), red-LEC and green-LEC (50:50), and green-LEC only. Representative images of CLS formation were imaged at 48 h. Scale bars are 500 *μ*m. CLS formed on 3D fibrin gel was quantified for tube length using AutoTube for (c) BEC and (d) LEC. Data represents mean $\pm$ SD, *n* = 4 per group, n.s. *p* > 0.05, *p < 0.05. All *P* values were determined by unpaired *t* tests. (e) An equal density of BEC or LEC (5 × 10^6^ cells/mL) were seeded on each chamber of the IdenTx Chips separated by the fibrin gels in the middle. Representative images of BEC or LEC sprouting into the fibrin gels after 48 h of culture. Scale bars are 500 *μ*m.

Both BECs and LECs have been shown to form cord-like structures (CLS) in 2D and 3D vasculogenesis assays.^23^ We previously used LECs to study *in vitro* lymphatic CLS induced by VEGF-C and substrate stiffness.^3^ Therefore, to investigate whether BECs and LECs can form capillary networks together or independent with respect to each other, we examined *in vitro* formation of CLS from co-cultures of BECs and LECs. We seeded BECs (pre-labeled in CellTracker™ Red CMTPX) and LECs (pre-labeled in CellTracker™ Green CMFDA) at ratios of 100:0, 80:20, 50:50, 20:80, and 0:100 (BECs:LECs) on 2D Matrigel (Fig. 2a and Supplementary Fig. 2). After 12 h, CLS formation was observed in all conditions. Interestingly, we found that BECs and LECs formed CLS independent of each other’s. We rarely found CLS that were formed by BECs and LECs together (Supplementary Fig. 2).

To further confirm this observation, we performed a 3D vasculogenesis assay using fibrin gels, where endothelial cells have been reported to form capillary networks with lumens.^12,14^ To quantify the separation of networks of LECs and BECs, we compared the network formation lengths of fibrin gels containing LECs and BECs of same color and cell type, different color but same cell type, and different color and cell type (Fig. 2b and Supplementary Fig. 3). After 48 h of encapsulation, the gels were imaged and the resulting fluorescent channels were quantified separately using MATLAB plugin AutoTube, which can quantify the skeletal length of vascular networks (Supplementary Fig. 4). Because LECs and BECs form intertwining networks when co-cultured *in vitro*, it is often difficult to visually confirm whether BECs and LECs form distinct networks. Using AutoTube, we confirmed that co-cultures of LECs and BECs labeled two different colors resulting in longer overall skeletal length on a single channel analysis compared to monocultures of LECs and BECs labeled two different colors (Fig. 2c). This indicates that unlike two-color monoculture, co-culture has continuous networks of cells of one color, which indicates that the LEC and BEC networks remain distinct. We also observed no significant difference between network lengths of co-culture and monoculture with same color (Fig. 2d), again indicating that LEC and BEC networks consist mostly of the same cell type even in co-cultures.

Since the cells were mixed indiscriminately before seeding onto the Matrigel and fibrin gel, it is impossible to completely prevent the inclusion of some LECs and BECs into the CLS of the other cell type. However, we observe that the cells do not elongate its morphology when embedded into the opposing CLS, which is a unique characteristic of angiogenic endothelial cells. While our AutoTube analysis shows that the two cell lines form distinct cord-like structures, due to the high density of cells necessary for meaningful AutoTube analysis, the separation of networks is not visibly clear. Therefore, we also performed the 3D angiogenesis assay in fibrin gels at half the cell density and observed visible distinct cord-like formation (Supplementary Fig. 3). We also performed an angiogenesis assay with IdenTX chip, where BECs and LECs were seeded along the media channels on the sides and allowed to invade the fibrin gel channel in the center. The cells formed networks across the gel channel only when same cell lines were seeded on both sides of the channel, indicating that the LECs and BECs form cord-like structures with the cells of the same type (Fig. 2e).

### Paracrine Signaling is Not Responsible for Distinct CLS Formation

**Figure 3:**
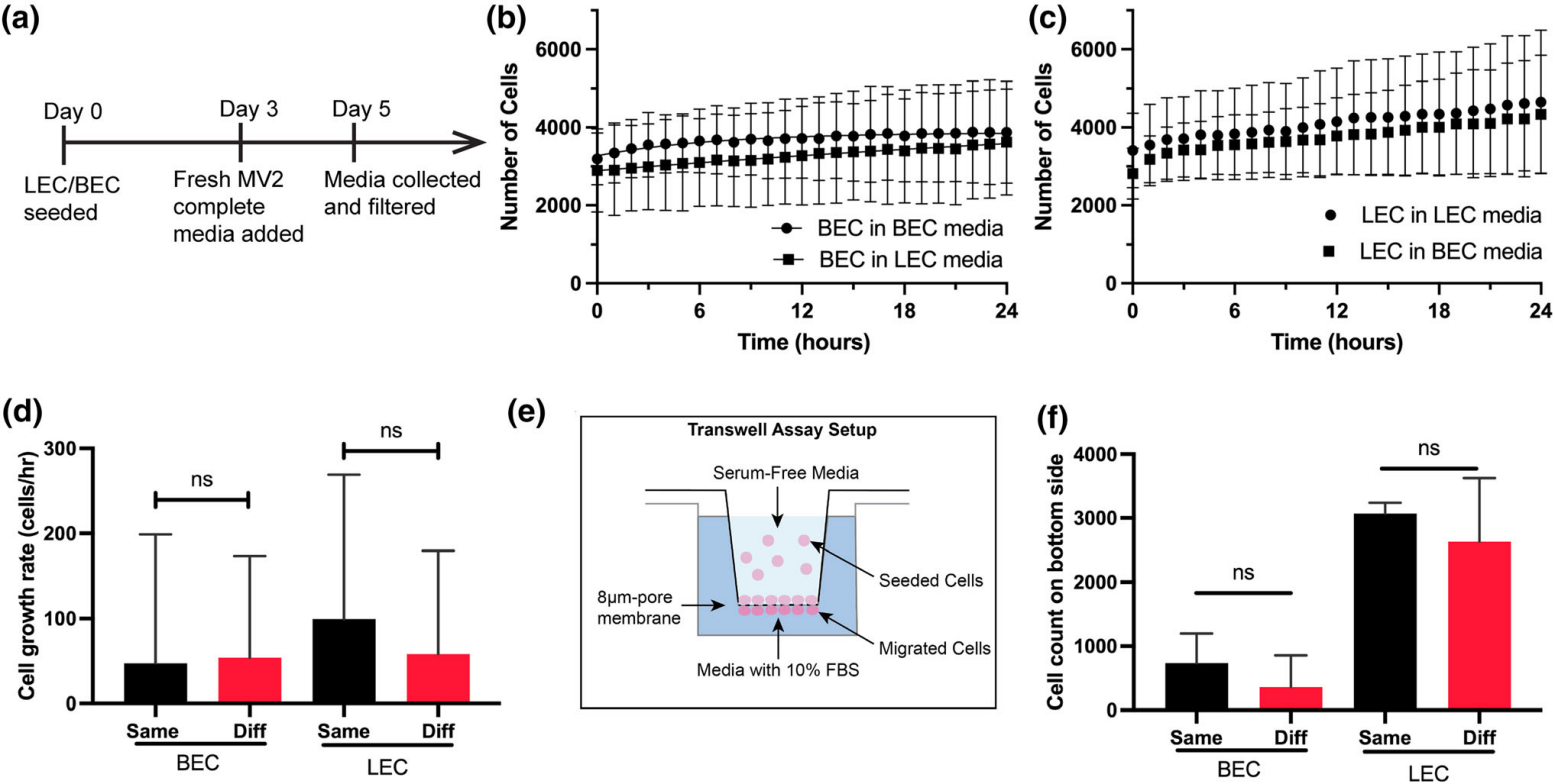
The effects of paracrine signaling on BEC and LEC. (a) A timeline for BEC and LEC-conditioned media collection. Conditioned media were collected after 48 h of BEC or LEC cultures. Cell proliferation assay with (b) BEC and (c) LEC using the same (circle data points) and different (square data points) cell line-conditioned media. (d) Cell growth rates of BEC and LEC were quantified at 3500 cells/well using the label-free cell proliferation protocol (Lionheart FX Microscope). Each sample was repeated 12 times. (e) A schematic diagram to illustrate the layout of the transwell migration assay. The outer well was filled with conditioned media from either the same or different cell type. (f) The top of the membrane was cleaned and imaged on a fluorescence channel to quantify the migrated cells after 24 h. Each sample was repeated 3 times. Data represents mean $\pm$ SD, *n* = 4 per group, n.s. *p* > 0.05, All *p* values were determined by unpaired *t* tests.

We then explored the possibility of paracrine signaling as a mechanism behind LEC-BEC recognition to form distinct CLS. LECs and BECs release a distinct set of paracrine signals which have been implicated in promoting growth of other tissues.^34,35^ To collect the paracrine signals, BECs and LECs were cultured in fresh MV2 media for 2 days, and the resulting media was collected and filtered (Fig. 3a). We performed cell proliferation assay of BECs and LECs when cultured in either conditioned media from BECs or LECs. We used label-free cell counting protocol of Lionheart FX Automated Microscope, which uses brightfield channel to count the number of cells at various timepoints (Figs. 3b and 3c). We compared the growth rate at around 3500 cells/well and found no significant differences between the two media types (Fig. 3d).

Furthermore, we performed a trans-well migration assay to test if the paracrine signals from BEC and LEC inhibit cell migration of the opposing cell line, where either media from the same or different cell line was placed in the outer well and cells were allowed to migrate across the bottom membrane (Fig. 3e). We found no significant differences in migration rate towards the media of same and different cell line, indicating that paracrine signaling is not responsible for affecting cell migration of BECs and LECs (Fig. 3f and Supplementary Fig. 5). Collectively, these results suggest that the BECs and LECs do not maintain CLS separation through paracrine signals. While it is possible that BECs and LECs respond to paracrine signals during angiogenesis, the BECs and LECs do not exhibit a different response that would indicate that paracrine signaling is primarily responsible for the distinct CLS formation.

### Podoplanin is Responsible for Distinct CLS Formation *In Vitro*

To determine if the recognition mechanism is through surface receptors, we tested the effect of Podoplanin on network separation. Previous studies have reported that Folliculin is responsible for LEC-BEC separation by downregulating *Prox1*, a master gene of LEC marker expression, in BECs.^54^ Since *PDPN* is one of the key markers of LEC, we hypothesized that BECs downregulate *PDPN* through *FLCN* expression, which allows BECs and LECs to recognize cells of the same lineage through membrane receptor *PDPN*. We generated LEC^Δ*PDPN*^ and BEC^Δ*FLCN*^ (denoted as ΔLEC and ΔBEC in the figures, respectively) through RNAi transfection. We performed quantitative PCR to confirm that RNAi-*PDPN* reduces *PDPN* expression by at least 90% in both LECs and BECs (Supplementary Fig. 6). RNAi-*FLCN* transfection increased *PDPN* expression in BECs by twofold but did not have significant result in *PDPN* expression in LECs, possibly due to already high levels of *PDPN* expression in LECs as confirmed in previous FACS data (Fig. 1g). We also quantified the basal levels of PDPN in LECs and BECs and determined that the twofold increase in PDPN would have biological significance.

**Figure 4:**
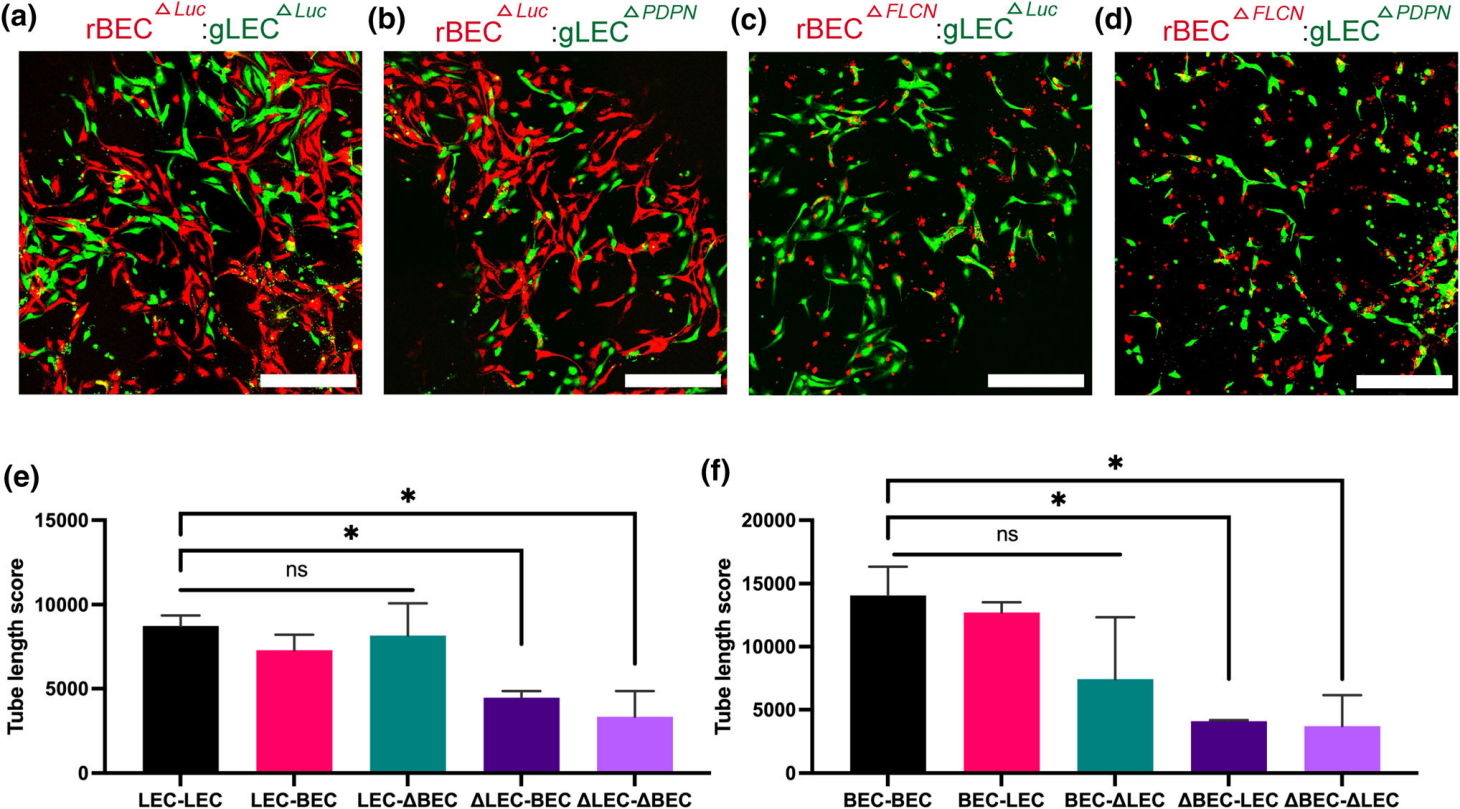
The roles of *FLCN* and *PDPN* on cord-like networks formation. (a and b) *Luciferase* knockout BEC (rBEC ^*ΔLuc*^) or (c and d) *FLCN* knockout BEC (rBEC^*ΔFLCN*^) stained in red are encapsulated in 3D fibrin gels together with (a and c) *Luciferase* knockout LEC (gLEC ^*ΔLuc*^) or (b and d) *PDPN* knockout LEC (rLEC^*ΔPDPN*^) stained in green. Representative images of CLS formation were imaged at 48 h. Scale bars are 500 *μ*m. (e and f) CLS formed on 3D fibrin gel was quantified for tube length using AutoTube. Data represents mean $\pm$ SD, *n* = 4 per group, n.s. *p* > 0.05, *p < 0.05. All *p* values were determined by ANOVA followed by *post hoc* testing with Tukey analysis.

Then, we performed vasculogenesis assay on 3D fibrin gels using LECs and BECs labeled with green or red membrane dyes. Each condition consisted of either green-labeled LEC or LEC^*ΔPDPN*^ and red-labeled BEC or BEC^*ΔFLCN*^ and imaged after 48 h under same conditions as previous fibrin gel assay (Figs. 4a–4d). The results were quantified using AutoTube as previously described, and the results were quantified along with LEC-LEC, BEC-BEC, and LEC-BEC data from before. We quantified LECs on the green channel and BECs on the red channel and measured the skeleton length of each network (Supplementary Fig. 4). We found that knockouts of LECs (Figs. 4b–4d) and BECs (Figs. 4a and 4d) failed to form networks by themselves and resulted in significantly shorter networks than same-color monoculture (Figs. 4e and 4f). To ensure that this observation is not due to reduced cell viability of knockout cells, we performed LIVE/DEAD viability assay and determined that siRNA transfected cells have viability of at least 90% (Supplementary Fig. 7). This indicates that the presence or lack of *PDPN* is crucial for BEC and LEC self-recognition and network formation. Comparatively, when wild-type cells were co-cultured with knockout cells, the network forming capabilities of the non-knockout cell line was not affected (Figs. 4e and 4f), which may indicate that these new knockout cells are also not being integrated into the network of the other cell line. We do not observe a significant difference between LEC^*ΔPDPN*^ and BEC when quantified using the green channel and BEC^*ΔFLCN*^ and LEC using the red channel, most likely because knockout cells generally have reduced ability to form networks resulting in shorter network lengths.

### Podoplanin is Responsible for the BEC and LEC Recognition

**Figure 5:**
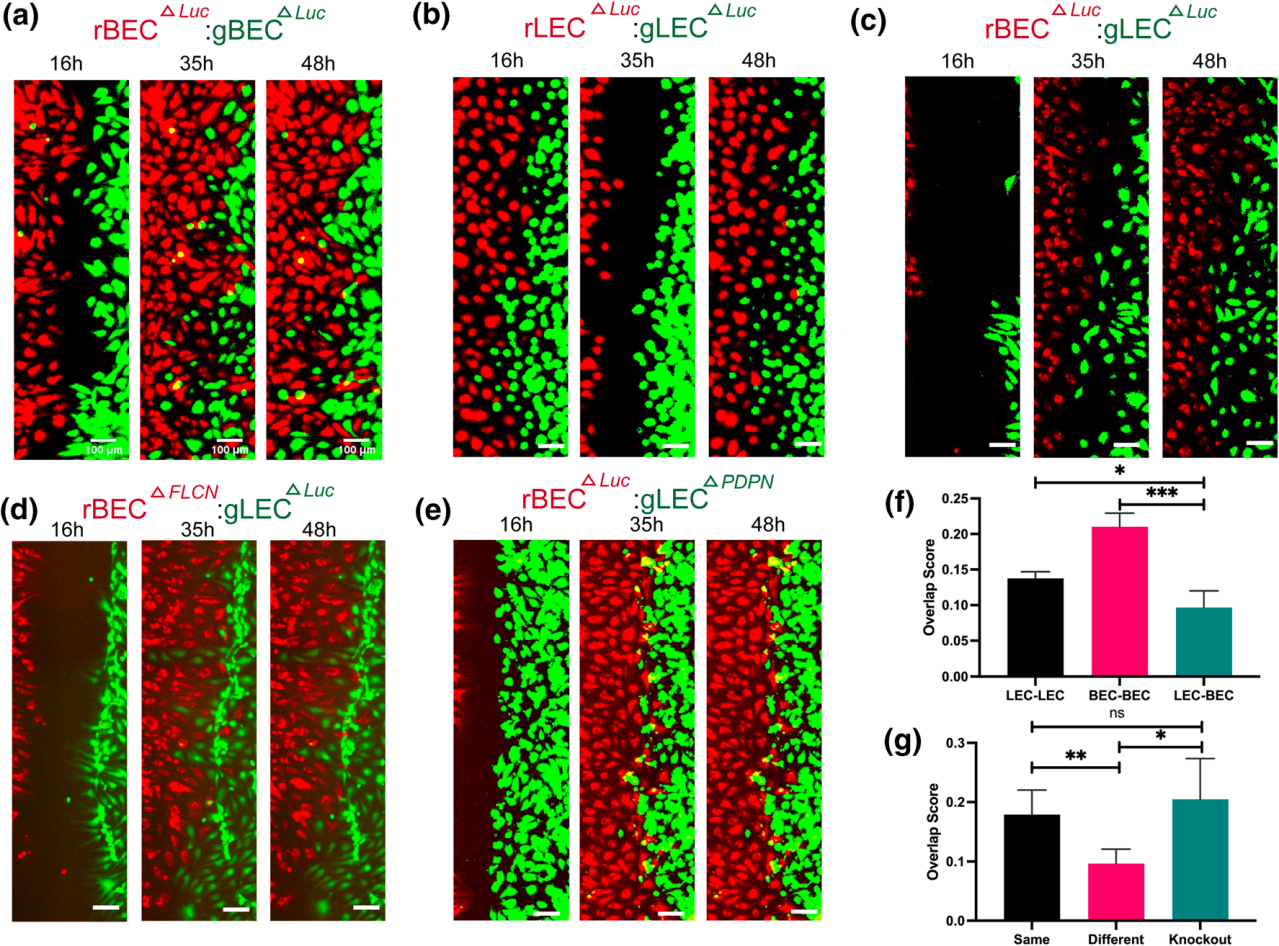
The roles of *FLCN* and *PDPN* on cell migration. Representative images of the wound healing assay taken at *t* = 16, 35, and 48 h for (a) BEC ^*ΔLuc*^ (in *red*): BEC ^*ΔLuc*^ (in *green*), (b) LEC ^*ΔLuc*^ (in *red*): LEC ^*ΔLuc*^ (in *green*), (c) BEC ^*ΔLuc*^ (in *red*): LEC ^*ΔLuc*^ (in *green*), (d) BEC^*ΔFLCN*^ (in *red*): LEC ^*ΔLuc*^ (in *green*), and (e) BEC^*ΔLuc*^ (in *red*): LEC ^*ΔPDPN*^ (in *green*). Each well was seeded with 7000 cells and allowed to settle for 24 h, then imaged for up to 48 h. Scale bars are 500 *μ*m. Overlap scores for (f) wild-type BEC and LEC, as well as (g) knock-out BEC and LEC. The LEC-BEC condition consists of 4 replicates and the same and knockout conditions consist of 4 replicates from either of the two conditions. Data represents mean $\pm$ SD, *n* = 4 per group, n.s. *p* > 0.05, *p < 0.05, **p < 0.01, and ***p < 0.001. All *p* values were determined by ANOVA followed by *post hoc* testing with Tukey analysis.

To fully confirm that LECs and BECs do not avoid BEC^*ΔFLCN*^ and LEC^*ΔPDPN*^ respectively, we performed wound healing assay to study how cell–cell contact affects cell migration for LEC and BEC. We used an ibidi two-well insert attached to a tissue culture plastic surface, with cells seeded on both wells stained with either the red or green membrane dyes. The conditions we tested were: LEC and LEC, BEC and BEC, LEC and BEC, BEC^*ΔFLCN*^ and LEC, and LEC^*ΔPDPN*^ and BEC (Figs. 5a–5e and Supplementary Movie 1–5). We seeded the cells and allowed them to adhere for 24 h, then we removed the insert and imaged the wound every hour for 48 h. The wound was fully closed around 24 h, and there was no significant difference in the wound closure time. However, we observed that in the LEC-BEC condition (Fig. 5c and Supplementary Movie 3), the cells appeared to change directions when first coming into contact with the other cell line, which resulted in a clear boundary between the two cell lines compared to BEC-BEC (Fig. 5a and Supplementary Movie 1) or BEC-BEC (Fig. 5b and Supplementary Movie 2) conditions.

We hypothesized that when BECs and LECs recognize the other cell line through presence or lack of *PDPN*, they change the direction of movement such that cells of the same lineage group together, which may be the mechanism behind how BECs and LECs form separate vessels *in vivo* even in close proximity with each other. To quantify this, we divided each segment of the wound into smaller segments and generated a density plot for the number of cells across the distance of the wound. Then we used the Overlap package in R to estimate the overlap scores in the two density plots. We observed that BECs and LECs do not mix in the middle, as shown by the lower overlap score (Fig. 5f). This effect is reversed when either the LEC or BEC is replaced with LEC^*ΔPDPN*^ or BEC^*ΔFLCN*^, which suggests that the knockouts of *PDPN* and *FLCN* in LEC and BEC, respectively, avoids being recognized as the different cell type by BEC and LEC (Fig. 5g). This indicates that the LEC-BEC recognition mechanism is through cell–cell contact through transmembrane protein *PDPN*.

## Discussion

Vascular tissue engineering is critical to the future of transplantable organ engineering as it has the potential to produce microvascular network that can overcome the diffusion limits in non-vascularized organoids.^13,21^ Consequently, multiple studies have focused on developing blood and lymphatic microvasculature networks in various hydrogels, including PEG and hyaluronic acid (HA)-hydrogels.^3,21,28,52^ Fibrin, a wound healing protein, is of particular interest in designing 3D hydrogels to promote vasculogenesis.^47^ Fibrin is compatible with recapitulating *in vivo* functionalities of both BECs and LECs and therefore has been used in microfluidic devices for LEC-BEC co-culturing to study how the two cell lines interact.^20,29^ Microvasculature engineering in organoids mostly relies on self-assembly of endothelial cells in hydrogels due to the extremely small size of these vessels, which relies on controlling molecular and biomechanical factors to direct network formation.^21,59^ To our knowledge, there are no studies thus far that explored the molecular mechanism behind LEC and BEC interaction in co-culture at a microvasculature level.

Multiple studies have explored the possible molecular pathways behind lymphatic and blood vessel separation pathways and identified numerous genes involved in this process,^10,17,45,55^ but none have identified a unifying pathway that explained how LECs and BECs only join vessels with the same type of cell during *in vivo* embryonic development or *in vitro* angiogenesis in hydrogels. In this study, we explored the molecular mechanism behind the widely reported BEC and LEC tendency to form distinct cord-like structures.^36^ Previous work has suggested the role of *PDPN* in preventing the mixing of the lymph and the blood through intermittent platelet aggregation near the lymphatic valve.^49,55^ *PDPN* is a well-conserved, mucin-type transmembrane protein that can interact with CLEC-2 expressing platelets.^9,45^ *FLCN* was also identified as a gene that maintains separation between the lymph and the blood through inhibition of *Prox1* in BECs.^54^ We have shown that microvasculature formation in fibrin gels by BECs and LECs can be manipulated through regulation of *FLCN* and *PDPN*. Our study suggests that *PDPN* plays a role in contact-based endothelial cell recognition and capillary formation in addition to lymphatic valve control.^40^ The change in direction of migration after coming into contact with the opposing cell line, as well as the reversal of this observation when *PDPN* is silenced in LECs or expressed in BECs, indicate that the cells recognize each other through contact-based mechanism via *PDPN*. Furthermore, we show that the downstream mechanism of *FLCN*-based LEC and BEC recognition is through the inhibition of *PDPN* expression, and that this LEC and BEC identity can be reversed by controlling *PDPN* or *FLCN* expression respectively.

Overall, our results indicate a novel cell–cell contact-based pathway for BECs and LECs to form distinct networks, which can be used to exert a higher degree of control over microvasculature assembly in vascular engineered tissues. This study suggests that FLCN and PDPN may be responsible for the BECs and LECs plasticity found in the zebrafish, rat mesentery, and human pluripotent stem cells.^41,48,54^ In general, blood capillaries are known to have tight junctions, while lymphatic capillaries display “button-like” structures with discontinuous and overlapping junctions.^3,21,32,53^ While CLS formed in Matrigel and fibrin gel were not able to fully capture these unique features, future studies could improve upon this finding by showing the lack of VE-Cadherin, which is responsible for cell–cell junctions in endothelial cells, between LECs and BECs forming CLS in the vicinity of each other to conclusively prove the lack of LEC-BEC network formation. In addition, future studies could further elucidate the role of PDPN in lymphangiogenesis during embryonic development and the initial separation of LECs from the blood vessels. In clinical applications, FLCN deficiency is associated with Birt-Hogg-Dubé syndrome and may additionally contribute to abnormal lymph nodes.^16,54^ These findings may lead to improved understanding of the effects of FLCN-related diseases on lymphatics, which may translate to novel therapeutic approaches to lymphatic disorders.

## Supplementary Information

Below is the link to the electronic supplementary material.

Supplementary file1 (MP4 605 KB)

Supplementary file2 (MP4 476 KB)

Supplementary file3 (MP4 269 KB)

Supplementary file4 (MP4 210 KB)

Supplementary file5 (MP4 792 KB)

Supplementary file6 (DOCX 19685 KB)

## Abbreviations

BECs: Blood endothelial cells
CLS: Cord-like structures
FLCN: Folliculin
LECs: Lymphatic endothelial cells
PDPN: Podoplanin
VEGF: Vascular endothelial growth factor
qRT-PCR: Real time quantitative reverse transcription polymerase chain reaction
