# Supplementary material for: Podoplanin is Responsible for the Distinct Blood and Lymphatic Capillaries
Source: Cell Mol Bioeng. 2022 Aug 6;15(5):467–78. doi: 10.1007/s12195-022-00730-2 (PMC9700554; doi:10.1007/s12195-022-00730-2)
Supplement: Supplementary file 6 — Supplementary file6 (DOCX 19685 KB) [file 12195_2022_730_MOESM6_ESM.docx]

**Supplementary Information**

**
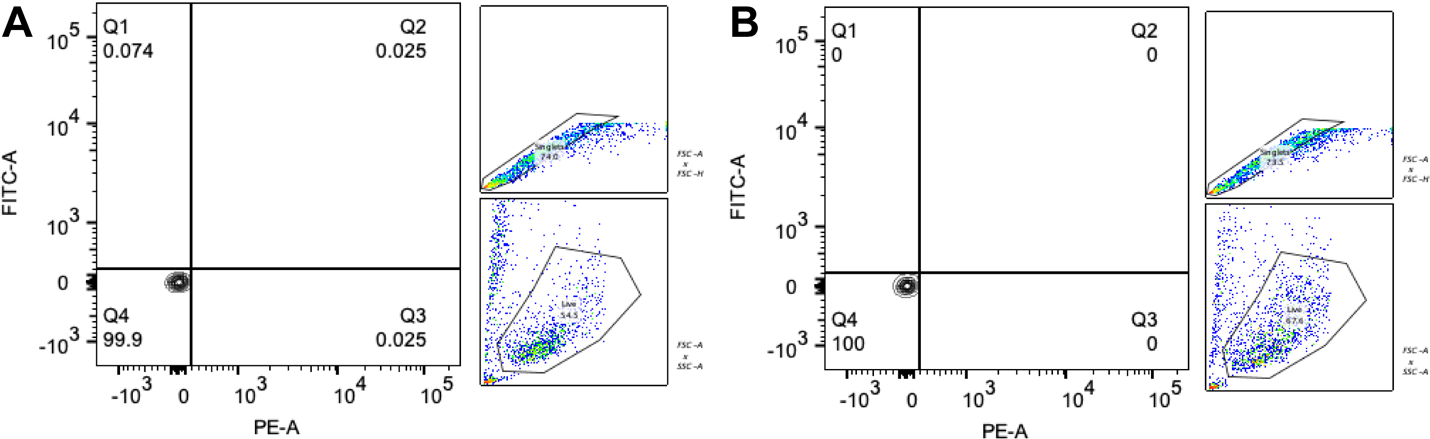
**

**Supplementary Figure 1. Flow cytometry analysis for BEC and LEC.** Representative flow cytometry diagrams demonstrating gating strategies for **(A)** BEC and **(B)** LEC stained with isotype controls.


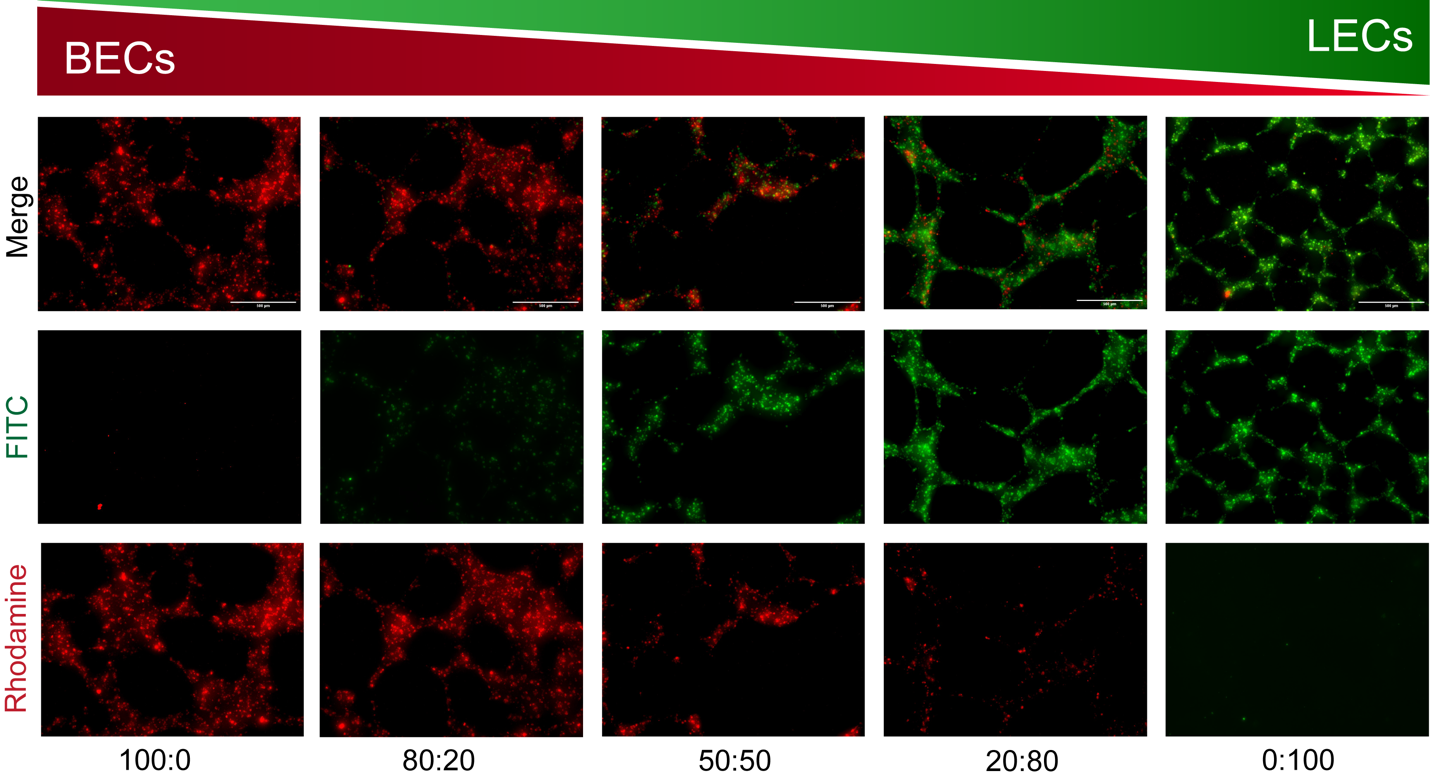


**Supplementary Figure 2. BEC and LEC form distinct cord-like structures on 2D Matrigel.** BEC (pre-labeled in pre-labeled in CellTracker™ Red CMTPX) and LEC (pre-labeled in CellTracker™ Green CMFDA) were seeded on 2D Matrigel at ratios of 100:0, 80:20, 50:50, 20:80, and 0:100 (BECs:LECs). Representative images of cord-like structures (CLS) formation were imaged at 12 hrs. Scale bars are 500 $\mu m$.

**
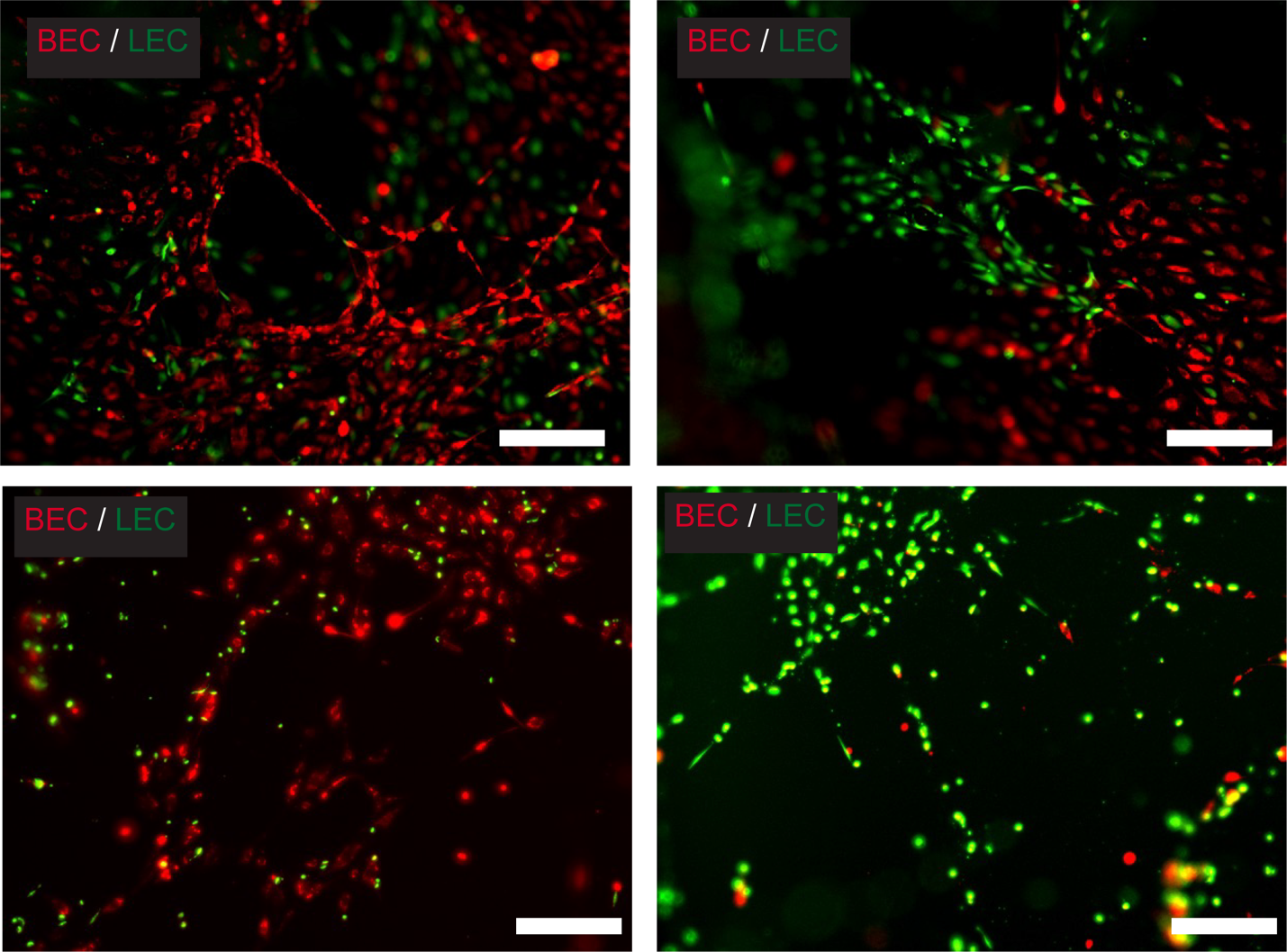
**

**Supplementary Figure 3. BEC and LEC form distinct cord-like structures on 3D fibrin gel assay.** BEC (pre-labeled in pre-labeled in CellTracker™ Red CMTPX) and LEC (pre-labeled in CellTracker™ Green CMFDA) were seeded on 3D fibrin gel. Representative images of cord-like structures (CLS) formation were imaged at 48 hrs. Scale bars are 250 $\mu m$.


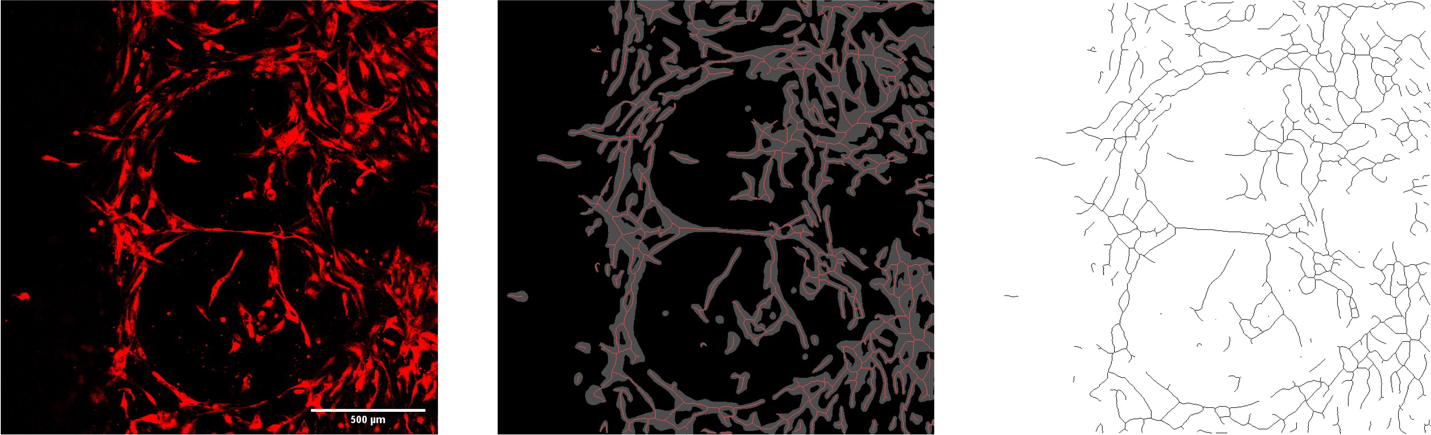


**Supplementary Figure 4.** Representative images to illustrate AutoTube quantification steps, the first image is the fluorescent image, second is identifying the cell boundaries, and the third is skeletonization of the network of cells.

**
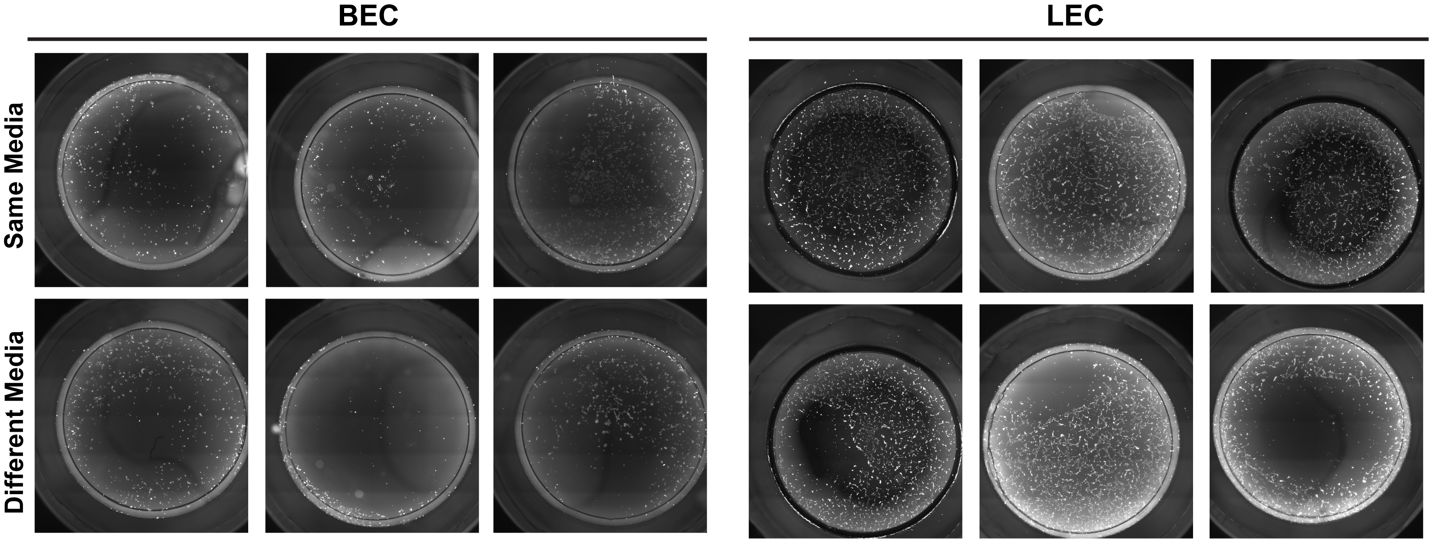
**

**Supplementary Figure 5. Transwell cell migration assay with BEC and LEC.** Representative images from the transwell cell migration assay for BEC and LEC with the same or different media. The top of the membrane was cleaned and imaged on a fluorescence channel to quantify the migrated cells after 24 hours. Each sample was repeated 3 times.


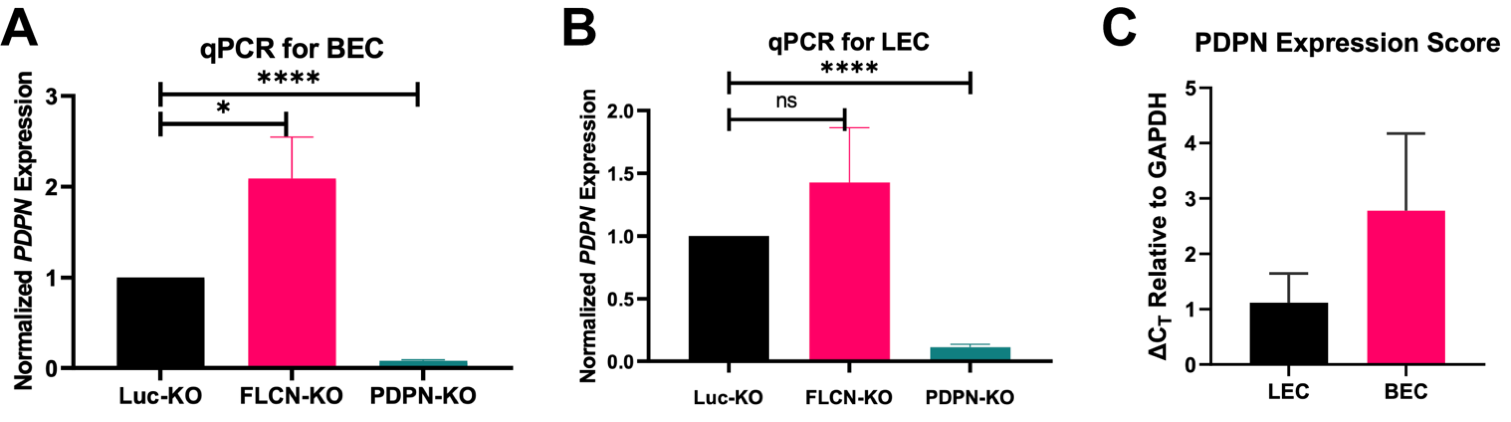


**Supplementary Figure 6.** Real-time quantitative RT-PCR to confirm knockout experiments in **(A)** BEC and **(B)** LEC. *PDPN* expression in control, *FLCN*, and *PDPN*-RNAi treated cells. Each were normalized to *PDPN* expression in Luciferase (non-targeting control). **(C)** *PDPN* expression score indicating $\Delta$C_T_ for *PDPN* expression relative to housekeeping gene GAPDH for LEC and BEC. Data represents mean $\pm$ SD, n=4 per group, n.s. *P* > 0.05, **P*<0.05, ***P*<0.01, and ****P*<0.001. All *P* values were determined by unpaired t tests.


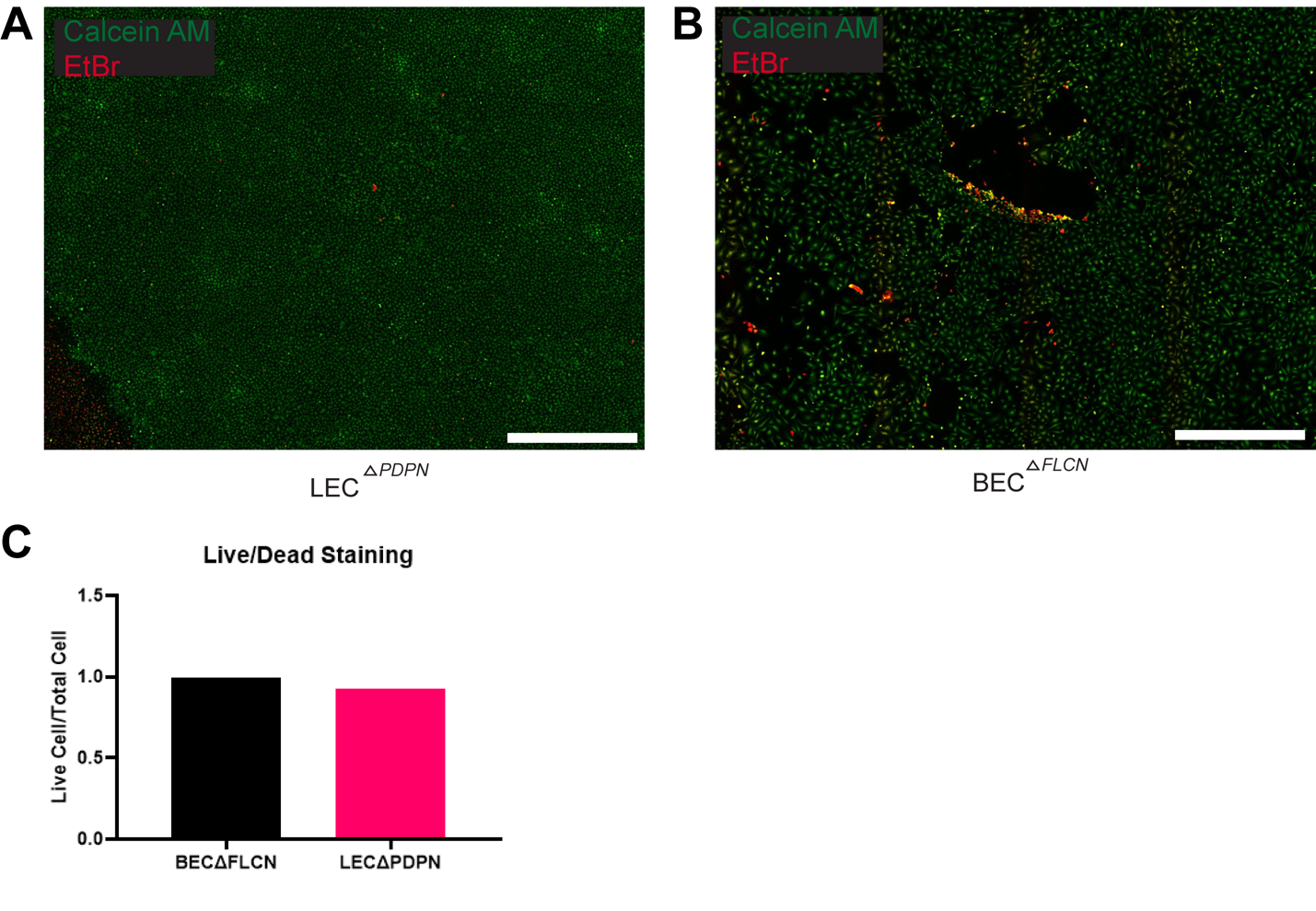


**Supplementary Figure 7. Cell viability of BEC and LEC following siRNA treatments.**  Representative fluorescent images of **(A)** BEC *^ΔFLCN^* and **(B)** LEC *^ΔPDPN^* stained with LIVE/DEAD assay at 48 hours following siRNA treatments. Scale bars are 1mm. **(C)** Quantification of fraction live cell normalized to total cell indicating more than 90% cell viability for BEC *^ΔFLCN^* and LEC *^ΔPDPN^*.

**Supplementary Movie 1. Cell migration assay for BEC (in *red*) : BEC (in *green*)**

**Supplementary Movie 2. Cell migration assay for LEC (in *red*) : LEC (in *green*)**

**Supplementary Movie 3. Cell migration assay for BEC (in *red*) : LEC (in *green*)**

**Supplementary Movie 4. Cell migration assay for BEC*^ΔFLCN^* (in *red*) : LEC (in *green*)**

**Supplementary Movie 5. Cell migration assay for BEC*^ΔFLCN^* (in *red*) : LEC (in *green*).**
